# Increased NADPH Supply Enhances Glycolysis Metabolic Flux and L-methionine Production in *Corynebacterium glutamicum*

**DOI:** 10.3390/foods11071031

**Published:** 2022-04-01

**Authors:** Bingnan Liu, Xinyu Sun, Yue Liu, Mengmeng Yang, Liang Wang, Ying Li, Jihui Wang

**Affiliations:** 1 School of Biological Engineering, Dalian Polytechnic University, Dalian 116034, China; lbnno158@foxmail.com (B.L.); sunxinyu0627@163.com (X.S.); sionliuyue@163.com (Y.L.); yangmm@dlyinyi.com (M.Y.); liangwang19862008@163.com (L.W.); 2 School of Food Science and Engineering, Dalian Ocean University, Dalian 116023, China; 3 Key Laboratory of Biotechnology and Bioresources Utilization, Dalian Minzu University, Dalian 116600, China; 4 Engineering Research Center of Health Food Design & Nutrition Regulation, School of Chemical Engineering and Energy Technology, Dongguan University of Technology, Dongguan 523808, China

**Keywords:** *Corynebacterium glutamicum*, glycolysis, L-methionine, metabolic flux, NADPH

## Abstract

*Corynebacterium glutamicum* is an important strain for the industrial production of amino acids, but the fermentation of L-methionine has not been realized. The purpose of this study is to clarify the effect of reducing power NADPH on L-methionine synthesis. Site-directed mutagenesis of *zwf* and *gnd* genes in pentose phosphate pathway relieved feedback inhibition, increased NADPH supply by 151.8%, and increased L-methionine production by 28.3%; Heterologous expression of *gapC* gene to introduce NADP^+^ dependent glyceraldehyde-3-phosphate dehydrogenase increased NADPH supply by 75.0% and L-methionine production by 48.7%; Heterologous expression of *pntAB* gene to introduce membrane-integral nicotinamide nucleotide transhydrogenase increased NADPH by 89.2% and L-methionine production by 35.9%. Finally, the engineering strain YM6 with a high NADPH supply was constructed, which increased the NADPH supply by 348.2% and the L-methionine production by 64.1%. The analysis of metabolic flux showed that YM6 significantly increased the glycolytic flux, including the metabolic flux of metabolites such as glycosyldehyde-3-phosphate, dihydroxyacetate phosphate, 3-phosphoglycate and pyruvate, and the significant increase of L-methionine flux also confirmed the increase of its synthesis. This study provides a research basis for the systematic metabolic engineering construction of L-methionine high-yield engineering strains.

## 1. Introduction

L-methionine is one of the eight essential amino acids that cannot be synthesized in human and animal bodies and is widely used in food, medicine, feed, and other industries [1,2,3]. However, L-methionine is still synthesized by chemical methods at present, and industrial microbial fermentation has not been realized [4]. The chemical synthesis of methionine mainly adopts the acrolein method [5]. Many harmful substances are produced in the synthesis process, and the synthetic product is DL-methionine, which needs further chiral resolution by aminoacylase [6]. In addition to the direct production of L-methionine, the microbial fermentation method also avoids the use of organic compounds that are unfavorable to the environment. The large-scale fermentation production of industrial amino acids such as glutamate and lysine has matured, providing the possibility for the industrial fermentation production of L-methionine.

*Corynebacterium glutamicum* is the main producer of the amino acid fermentation industry in the world, such as glutamate, lysine, and threonine [7]. In order to realize the industrialized fermentation production of L-methionine, its production cost is required to be comparable to that of chemical synthesis, but the current yield is far from meeting the requirements. After systematic metabolic engineering, the current maximum production of L-methionine can only reach about 6 g/L [8,9]. Therefore, it is necessary to clarify the regulation mechanism of L-methionine and find new metabolic engineering targets. One of the reasons why L-methionine is more difficult to synthesize by *C. glutamicum* compared with other amino acids is that the structure of L-methionine contains sulfur atoms, and the inorganic sulfur source needs to be reduced before it can be used for L-methionine synthesis, which requires more reducing power [10]. NADPH plays a major reducing role in cells. It takes 1 mol and 4 mol of NADPH to produce 1 mol of L- glutamate and L-lysine, respectively [11], while the NADPH is required to produce 1 mol of L-methionine is 8 mol [9]. Therefore, overproduction of L-methionine requires more NADPH supply.

**Figure 1:**
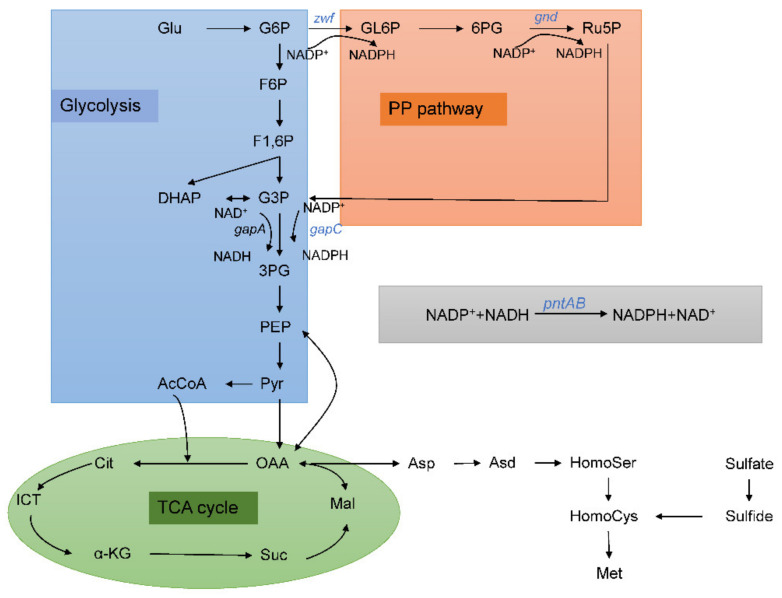
Central metabolic pathways and NADPH generation reactions in L-methionine production. The genes represented in blue color are modified in this study. Abbreviations: Glu, glucose; G6P, glucose-6-phosphate; F6P, fructose-6-phosphate; F1,6BP, fructose-1,6-bisphosphate; G3P, glyceraldehyde-3-phosphate; 3PG, 3-phosphoglycerate; PEP, phosphoenolpyruvate; Pyr, pyruvate; AcCoA, acetyl coenzyme A; GL6P, gluconolactone-6-phosphate; 6PG, 6-phosphogluconate; Ru5P, ribulose-5-phosphate; OAA, oxaloacetate; Cit, citrate; ICT, isocitrate; a-KG, a-ketoglutarate; Suc, succinate; Mal, malate; Asp, aspartate; Asd, aspartate semialdehyde; HomoSer, homoserine; HomoCys, homocysteine; Met, methionine.

In *C. glutamicum*, NADPH is mainly derived from the two-step dehydrogenation reaction in the pentose phosphate pathway, that is, the step of generating gluconolactone-6-phosphate from glucose-6-phosphate and the step of generating ribulose-5-phosphate from 6-phosphogluconate (Figure 1). However, too much NADPH will inhibit the above two reactions, so enzyme modification to remove the feedback inhibition is an important step to increase the intracellular NADPH content [12]. In the reaction of synthesizing 3-phosphoglycerate from glyceraldehyde-3-phosphate, glyceraldehyde-3-phosphate dehydrogenase belongs to the NAD^+^-dependent type and cannot synthesize NADPH. Heterologous expression of *gapC* from *Clostridium acetobutylicum* can introduce NADP^+^-dependent glyceraldehyde 3-phosphate dehydrogenase, thereby increasing NADPH supply [13]. In addition, the heterologous expression of nicotinamide nucleotide transhydrogenase in *E. coli* can also promote the conversion of NADP^+^ to NADPH [14]. Therefore, this work used the above methods to study the effect of increased NADPH supply on L-methionine synthesis in *C. glutamicum* and studied the change of central carbon metabolism after increased NADPH supply through ^13^C metabolic flux analysis, to provide some theoretical reference for the construction of L-methionine engineering strains.

## 2. Materials and Methods

### 2.1. Bacterial Strains and Culture Conditions

*E. coli Top10* and *C. glutamicum* ATCC13032 strains were routinely cultured on LB medium (yeast extract 5 g/L, peptone 10 g/L, NaCl 10 g/L). LBHIS medium (yeast extract 2.5 g/L, peptone 5 g/L, NaCl 5 g/L, brain heart infusion broth 18.5 g/L, glycine 91 g/L) and LBS medium (LB medium supplemented with 10 g/L sucrose) were used for two screens after transformation of *C. glutamicum*. Epo medium (yeast extract 5 g/L, peptone 10 g/L, NaCl 10 g/L, Glucose 5 g/L, glycine 25 g/L, Isoniazid 4 g/L) was used for the preparation of competent cells of *C. glutamicum*. CGXII minimal medium [15] for L-methionine fermentation. Solid media were supplemented with 2% agar powder. *E. coli* was incubated at 37 °C, and *C. glutamicum* was incubated at 30 °C. Appropriate concentrations of antibiotics were added as needed during the incubation. The microbial strains and plasmids used in this study and their properties are shown in Table 1.

### 2.2. Primers and Plasmids

The primer sequences used in this experiment are shown in Table 2. Plasmid pK18mobsacB and pLY4 were used in this work [9,16] and are shown in Table 1.

### 2.3. Site-Directed Mutation of zwf and gnd Gene

**Figure 2:**
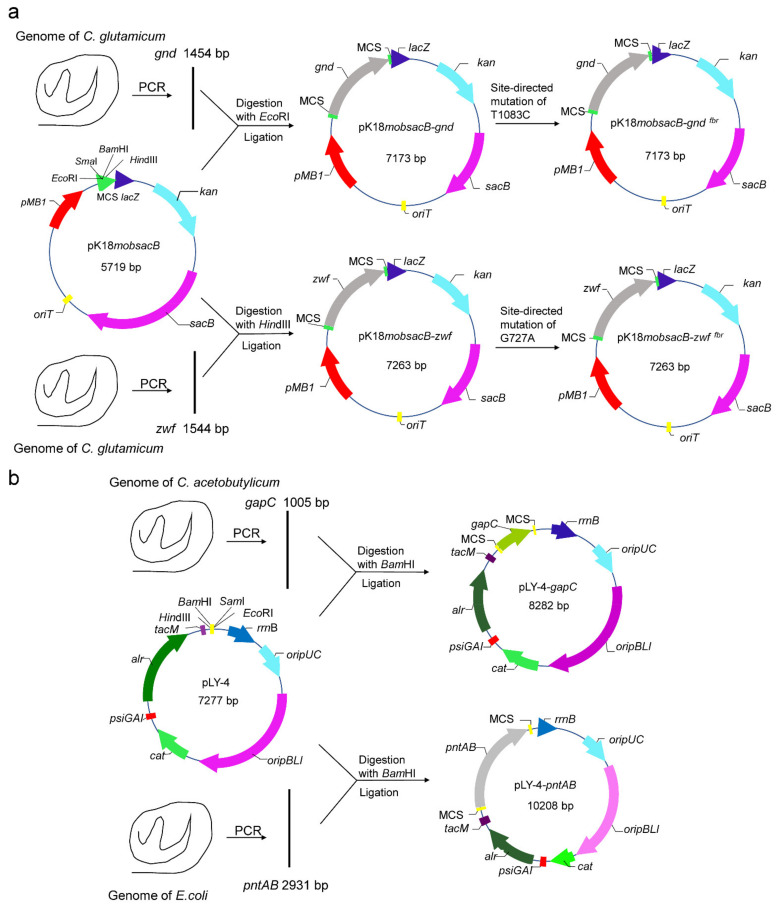
Plasmid construction. (**a**) Site-directed mutation plasmids of *gnd* and *zwf*; (**b**) Heterologous expression plasmid of *gapC* and *pntAB*.

The Fast Mutagenesis System (TransGen Biotech Co., Ltd., Beijing, China) was used for site-directed mutation, and the protocols were described as follows briefly. Using primers zwf-F/gnd-F and zwf-R/gnd-R, the *zwf*/*gnd* gene was amplified from the genome of *C. glutamicum*. After digestion with *Hin*dIII/*Ero*RI and ligation, the *zwf*/*gnd* gene was ligated to pK18mobsacB to obtain pK18mobsacB-zwf/pK18mobsacB-gnd. The recombinant plasmid (1–10 ng) was used as the template for PCR reaction with mutation primers (zwf-TBF and zwf-TBR/gnd-TBF and gnd-TBR) to amplify the recombinant plasmid. 10 μL of the PCR product was taken for electrophoretic detection. 1 μL of DMT enzyme was added to the remaining PCR product, mixed thoroughly, and incubated at 37 °C for 1 h. 10 μL of the digested product was taken for the transformation of DMT receptor cells for the final screening of the mutant plasmid. The mutant plasmid was sent to BGI Genomics (Beijing) for sequencing. The construction process of the plasmid was shown in Figure 2a.

### 2.4. Construction of Expression Vectors pLY-4-gapC and pLY-4-pntAB

Using *C. acetobutylicum/E. coli* genome as the template, *gapc*/*pntAB* gene was amplified by PCR with the primers gapc-F and gapc-R/pntAB-F and pntAB-R. The plasmid pLY-4 and the *gapc*/*pntAB* gene were digested with *Bam*HI, ligated, and transformed into *E. coli* Top10. The transformants were screened and the plasmid pLY-4-*gapC*/pLY-4-*pntAB* was obtained. The construction process of the plasmid was shown in Figure 2b.

### 2.5. Transformation Method

Molecular cloning and transformation of *E. coli Top10* cells were performed using standard procedures [17]. The electroshock transformation method of the receptor cells of *C. glutamicum* was based on the method established by van der Rest et al., with slight modifications [18]. Take a tube of *C. glutamicum* receptor cells, add 10 μL of recombinant plasmid, and mix thoroughly. The above bacterial solution was transferred to a pre-cooled shock cup on ice for 5 min and shocked twice consecutively at 1800 V for 5 ms. 1mL of pre-warmed LBHIS liquid medium was quickly added to the shock cup and placed in a 46 °C water bath for 6 min of heat excitation, followed by 30 °C, 180 rpm shaking to recover the culture for 2 h. The above bacterial solution was collected by centrifugation at 5000× *g* and coated with LBHIS solid plates containing 25 μg/mL of kanamycin (for site-directed mutation) or chloramphenicol (for heterologous expression) and incubated at 30 °C for 4 days to screen the transformants.

### 2.6. Determination of NADPH

About 1 × 10^6^ cells were collected and washed with PBS buffer three times, then ground with liquid nitrogen to break the cell walls. Intracellular NADPH content was determined using the NADP^+^/NADPH Detection Kit of Beyotime Biotechnology Co., Ltd., Shanghai, China.

### 2.7. Determination of L-methionine

The content of L-methionine was determined by a special chromatographic column system for amino acid analysis (including a column, a guard column, an amino acid kit, and amino acid standard; Dalian Elite Analytical Instrument Co., Ltd., Dalian, China) on high-performance liquid chromatography (HPLC; e2695, Waters, USA). After 5 mL of the fermentation broth was centrifuged at 5000× *g* for 10 min, the supernatant was added with 5% trichloroacetic acid, mixed well, and left to stand for precipitation, and the supernatant was taken and filtered with a 0.45 μm membrane. 1 mL of derivatization reagent in the amino acid kit was added to 2 mL of the filtered sample in a brown bottle and maintained in the dark at 60 °C for 1 h. After the reaction was cooled to room temperature, the equilibrium buffer in the amino acid kit was added to 10 mL and left for 15 min. After filtering with a 0.45 μm membrane, the sample was ready for HPLC detection [19].

### 2.8. Gene Expression Analysis

After centrifugation at 10,000× *g* for 5 min, the cells were collected and fully ground with liquid nitrogen. RNAiso plus (Takara Biomedical Technology Co., Ltd., Beijing, China) was used for total RNA extraction, and the operation was carried out according to the method in the manual. Transcript ^®^ Green one-step qRT PCR Supermix (TransGen Biotech Co., Ltd., Beijing, China) was used for qRT-PCR. The relative expression was calculated by the 2^−^^△△ct^ method.

### 2.9. ^13^C-Metabolic Flux Ratio of Metabolites from the Labeled Glucose

The ^13^C labeling experiments were combined with GC-MS chromatographic information to analyze the ^13^C labeling status of intracellular intermediate metabolites, thereby systematically quantifying the relative magnitude of individual metabolic fluxes and their distribution changes within cells.

Wild-type *C. glutamicum* 13,032 and *C. glutamicum* YM6 were cultured to OD_600_ = 0.8 using ^13^C-labeled glucose (D-Glucose-^13^C_6_, 99%, Cambridge Isotope Laboratories, Tewksbury, Massachusetts, USA) as the sole carbon source in CGXII medium. The cells were collected by centrifugation at 10,000× *g*, 4 °C for 10 min, and quenched by rapid immersion in liquid nitrogen for 30 s. After thawing on ice, the residual culture medium was washed with PBS buffer, and then the PBS buffer was removed by centrifugation at 1000× *g*, 4 °C, for 10 min. The cells were resuspended with 600 μL of 50% cold methanol solution and internal standard and inserted into dry ice for 30 min, then thawed at 4 °C. 400 μL of chloroform was added to the sample, shaken for 30 s, and centrifuged at 12,000× *g* and 4 °C for 10 min. The upper layer of the solution was freeze-dried. After that, 70 μL of pyridine was added, mixed well, and reacted at 80 °C for 20 min. 30 μL of derivatization reagent (TBDMS) was continued to be added and then derivatized at 80 °C for 1 h. After centrifuging at 10,000× *g* for 10 min at 4 °C, the supernatant was transferred to the test vial of GC-MS for testing. A Shimadzu QP-2010 Ultra GC-MS was programmed with an injection temperature of 250 °C injections and injected with 1 µL of the sample. GC oven temperature started at 110 °C for 4 min, rising to 230 °C at 3 °C/min and to 280 °C at 20 °C/min with a final hold at this temperature for 2 min. GC flow rate with helium carrier gas was 50 cm/s. The GC column used was a 20 m × 0.25 mm × 0.25 mm Rxi-5 ms. GC-MS interface temperature was 300 °C and (electron impact) ion source temperature was set at 200 °C, with 70 V ionization voltage. The mass spectrometer was set to scan *m*/*z* range 50–800, with a 1 kV detector.

### 2.10. Data Processing and Statistical Analysis

For GC-MS data, peak maps and isotopic information of metabolites from GC-MS were collected, and then Matlab flux-8, a professional metabolic flow analysis software based on Matlab foundation, was used to analyze the isotopic information of obtained metabolites, and the isotopic distribution information of each compound was directly calculated with the ^13^C flux ratio of each metabolite from the labeled carbon source in the extracellular and intracellular pathways to further compare the changes in the experimental and control groups.

All experiments consisted of three replicates. The results were expressed as the means ± SD, and analyses were based on one-way ANOVA, followed by the Fisher PLSD post hoc test if differences were significant. The limit of statistical significance was set at *p* < 0.05.

## 3. Results

### 3.1. Modification of Pentose Phosphate Pathway to Increase NADPH Supplement

The pentose phosphate pathway is the major intracellular metabolic pathway for the production of reducing NADPH, glucose-6-phosphate dehydrogenase (encoded by the *zwf* gene), and 6-phosphogluconate dehydrogenase (encoded by the *gnd* gene) are the key enzymes this pathway. In this study, G at site 727 of *zwf* gene was mutated to A, and T at site 1083 of *gnd* gene was mutated to C to release the feedback inhibition of glucose-6-phosphate dehydrogenase and 6-phosphogluconate dehydrogenase respectively, thereby increasing intracellular NADPH supply. Plasmid pK18mobsacB-zwf^fbr^ and pK18mobsacB-gnd^fbr^ were transformed into *C. glutamicum* 13032, and *C. glutamicum* YM1 and YM2 were obtained, respectively. The plasmid pK18mobsacB-gnd^fbr^ was transformed into *C. glutamicum* YM1 to obtain *C. glutamicum* YM3. In order to determine whether *C. glutamicum* YM1, YM2, and YM3 have changed the intracellular NADPH level, the intracellular NADPH contents of the original strain *C. glutamicum* and the mutant strains were measured (Table 3). Compared with the wild-type strain, the NADPH content of *C. glutamicum* YM1, YM2, and YM3 was increased by 67.9%, 57.1%, and 151.8%, and the L-methionine production was increased by 13.53%, 16.32%, and 28.3%, respectively. The simultaneous site-directed mutation of *zwf* and *gnd* resulted in a large increase in NADPH content, but no significant increase in L-methionine production compared with mutation of one gene alone. In addition, through gene expression analysis, it was found that the expression of *zwf* and *gnd* genes in *C. glutamicum* YM1, YM2, and YM3 did not change compared with the wild-type strain, indicating that the increase of NADPH content by site-directed mutation did not change the gene expression but relieved the feedback inhibition of the enzyme.

### 3.2. Heterologous Expression of gapC and pntAB

**Figure 3:**
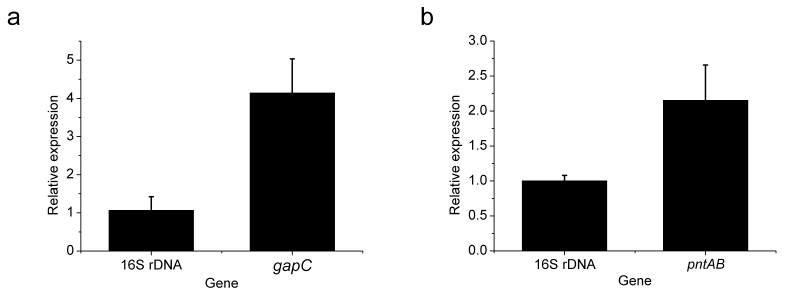
Expression of *gapC* in strain YM4 (**a**) and *pntAB* in strain YM5 (**b**). The 2^−10^ of the 16S rDNA gene expression level in each strain was set as 1.

The glyceraldehyde-3-phosphate dehydrogenase (encoded by the gene *gapC*) in *C. acetobutylicum* is primarily NADP^+^-dependent, which catalyzes glyceraldehyde-3-phosphate and NADP^+^ to form 3-phospho-D-glyceroyl phosphate and NADPH. Due to the lack of NADP^+^-dependent glyceraldehyde-3-phosphate dehydrogenase in *C. glutamicum*, heterologous expression of *gapC* can increase the intracellular NADPH supply. The membrane-integral nicotinamide nucleotide transhydrogenase (encoded by the gene *pntAB*) in *E. coli* can drive the reduction of NADP^+^ to NADPH via the oxidation of NADH to NAD^+^. As *C. glutamicum* does not possess such an enzyme, the expression of *pntAB* is helpful to increase the supply of NADPH. We heterologously expressed *gapc* and *pntAB* genes in *C. glutamicum* 13,032, respectively, to obtain strains *C. glutamicum* YM4 and YM5. As seen in Table 3, the expression of both genes significantly increased NADPH supply and L-methionine synthesis. The L-methionine production in *C. glutamicum* YM4 and YM5 reached 0.58 g/L and 0.53 g/L, which were 48.7% and 35.9% higher than those in the wild-type strain, respectively. Further gene expression analysis revealed that the increase in NADPH was caused by heterologous expression of *gapC* or *pntAB*, but not the *zwf* or *gnd* genes (Figure 3).

### 3.3. Construction and Fermentation Characteristics of C. glutamicum YM6

**Figure 4:**
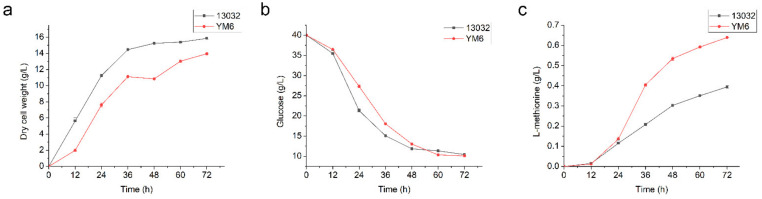
Dry cell weight (**a**), glucose consumption (**b**), and methionine production (**c**) during batch fermentation of *C. glutamicum* strains ATCC13032 (represented by squares) and YM6 (represented by circles).

*C. glutamicum* YM6 was obtained by expressing the *gapC* gene on the basis of *C. glutamicum* YM3. As seen in Table 3, the NADPH content of *C. glutamicum* YM6 was 2.51 nmol/10^6^ cell, which was 78.0% higher than that of *C. glutamicum* YM3, and 348.2% higher than that of the wild-type strain. Meanwhile, the L-methionine yield of *C. glutamicum* YM6 reached 0.64 g/L, which was 28.0% higher than that of *C. glutamicum* YM3 and 64.1% higher than that of the wild-type strain. Figure 4 showed the fermentation characteristics of *C. glutamicum* YM6 and *C. glutamicum* 13,032, including glucose consumption, biomass accumulation, and L-methionine production. In the cell growth, the accumulation of biomass in *C. glutamicum* YM6 was inhibited (from 15.86 g/L to 13.96 g/L), showing the characteristics of a long lag phase and a short logarithmic phase. This reduced the cell yield Y_X/S_ by 13.3% (from 0.52 g/g to 0.45 g/g) compared with the wild-type strain but increased the maximum specific growth rate μ_max_ by 84.6% (from 0.039 h^−1^ to 0.072 h^−1^). In L-methionine production, the product yield Y_P/S_ of *C. glutamicum* YM6 was 0.021 g/g, while that of the wild-type strain was 0.013 g/g.

### 3.4. Metabolic Flux Ratio Analysis of C. glutamicum YM6

**Figure 5:**
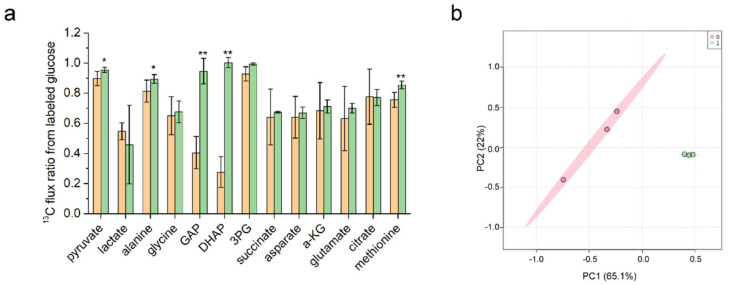
(**a**) Differences in ^13^C flux ratio between strain YM6 (orange) and the wild-type strain (green) in glycolytic. * indicates a significant difference between the two groups (*p* < 0.05), ** indicates a significant difference between the two groups (*p* < 0.01). (**b**) Comparative analysis of the ^13^C flux ratio component differences between the strain YM6 (green) and the wild-type strain (pink) using principal component analysis.

The ^13^C labeling state of the intracellular intermediate metabolites was analyzed by the ^13^C labeling experiment and GC-MS chromatographic information, to calculate the relative changes in the ^13^C flux ratio of metabolites of the intracellular metabolic pathways. As shown in Figure 5a, comparing *C. glutamicum* 13,032, the flux ratio of multiple metabolites in the glycolytic pathway was significantly increased in *C. glutamicum* YM6, such as glyceraldehyde-3-phosphate, dihydroxyacetone phosphate, 3-phosphoglycerate, and pyruvate. The L-methionine flux ratio increased significantly, indicating that more glucose entered the L-methionine pathway. Through PC component analysis (Figure 5b), the difference in ^13^C flux ratio components between the two groups was compared, and it was found that the metabolic flux distribution characteristics of the two groups could be separated independently by component classification, indicating that significant metabolic remodeling occurred in *C. glutamicum* YM6 after modification.

## 4. Discussion

In the past few decades, although researchers have done a lot of work in the breeding and selection of L-methionine-producing strains, they still cannot meet the needs of industrial production [8,9]. This may be related to the synthesis of L-methionine, which is a sulfur-containing amino acid that is regulated by sulfur metabolism. The +6-valent sulfate group needs to be reduced to −2-valent sulfide before it can be used to synthesize amino acids. Studies have used reduced methanethiol and dimethyl disulfide as the only sulfur source and found that *C. glutamicum* was able to grow. However, due to a limited supply of sulfur for cysteine synthesis and the toxicity of high concentration sulfide, the specific growth rate of cells decreased significantly [20]. Therefore, as the main source of reducing power in biosynthesis, NADPH is a key to sulfate reduction. Engineering NADPH synthesis pathways have been widely used in amino acid biosynthesis. Wang et al., constructed a high intracellular NADPH level of the *C. glutamicum* strain, compared with the starting strain, the production of L-lysine was increased by 1.86-fold, and the production of L-leucine was increased by 1.4-fold [12]. In this study, strain YM6 with site-directed mutation of *zwf* and *gnd*, as well as heterologous expression of *gapC* showed an increased intracellular NADPH level (increased by 448.2%). The production of L-methionine in YM6 increased by 64.1%, however, it showed a low dry cell weight. This may be caused by heterologous expression or an imbalance of the redox state. Unbalanced NADPH may inhibit the yield of products [21].

Since NADPH is the main source of reducing power in biosynthesis, and its deficiency has become a major limiting factor in the production of amino acids such as lysine, how to increase the supply of intracellular NADPH to meet the production demand has become a focus of research [12,22]. In addition, because NADPH is involved in many enzymatic reactions in intracellular biosynthetic pathways, an unsustainable supply of NADPH not only directly affects the biosynthesis of amino acids but also affects cellular functions and cell survival [22]. In L-methionine biosynthesis, the unsustainable supply of NADPH is particularly serious, because 8 mol of NADPH is required to synthesize 1 mol of L-methionine, which is much more than that required for the synthesis of other amino acids [10]. The main source of NADPH in *C. glutamicum* is the pentose phosphate pathway, and *zwf* and *gnd* are key genes providing NADPH [23]. Therefore, many researchers have used overexpression, promoter modification, and site-directed mutation to increase PPP pathway flux and NADPH supply [14,24]. To allow more carbon flux to enter the PPP pathway and thus provide more NADPH, a good option would be to relieve the feedback inhibition of G6PDH. Studies have shown that mutating the G at 727 of the *zwf* gene to an A could reduce sensitivity against inhibition by ATP, phosphoenolpyruvate, and fructose 1,6-bisphosphate, which can increase intracellular NADPH and effectively increase the production of L-lysine [25,26]. Ser-361→Phe mutation in *gnd* reduced allosteric inhibition by intracellular metabolites, such as fructose 1,6-bisphosphate, D-glyceraldehyde 3-phosphate, phosphoribosyl pyrophosphate, ATP, and NADPH [27]. In addition, since there is only NAD^+^-dependent glyceraldehyde 3-phosphate dehydrogenase in *C. glutamicum*, the addition of NADP^+^-dependent glyceraldehyde 3-phosphate dehydrogenase from *C. acetobutyricum* can increase the NADPH generation pathway, resulting in an increase in the intracellular NADPH supply [13]. Expression of *pntAB* from *E. coli* can directly reduce the accumulation of intracellular NADH and increase the supply of NADPH [14].

In the study, it was found that the simultaneous mutation of *zwf* and *gnd* (strain YM3) can greatly increase the NADPH supply but compared with the single mutation (strain YM1 or YM2), L-methionine production did not increase significantly. Heterologous expression of both *gapC* and *pntAB* significantly increased L-methionine production. But strain YM6 only increased L-methionine production by 28% compared to YM3, while NADPH supply increased by 78%. These results all indicate that increasing the supply of NADPH does increase the production of L-methionine, but the excess NADPH is also used for the synthesis of metabolites such as other amino acids. Therefore, while increasing the supply of NADPH, systematic metabolic engineering is necessary for the construction of L-methionine engineered strains. In addition, NADPH imbalance sometimes affects product synthesis [28], rational control of NADPH levels may help to increase L-methionine synthesis.

Fermentation analysis showed that increased NADPH supply significantly increased the conversion rate of glucose to L-methionine, suggesting that the central carbon metabolic flux may be reconstructed. Previous studies have shown that *zwf* and *gnd* gene mutations significantly enhance PPP pathway flux [25,27,29], so we further studied the changes in metabolites flux ratio in glycolysis and TCA pathway. Overall, the *C. glutamicum* YM6 glycolytic flux was significantly increased compared to the wild-type strain, while the flux of TCA did not change significantly. Among them, GAP and DHAP changed the most, which may be caused by the increased flux of the PPP pathway. GAP and DHAP, two natural triose phosphate, serve as metabolic hubs linking glycolysis and PPP pathways [30]. At the same time, as substrates for the reaction catalyzed by glyceraldehyde-3-phosphate dehydrogenase, they also provide the possibility for the synthesis of more NADPH. Pyruvate is an important precursor of L-methionine synthesis, and its flux ratio increase is beneficial to L-methionine synthesis. The significant increase of alanine flux further indicates that excess NADPH will also be used for the synthesis of other amino acids. Increasing NADPH supply significantly increased the metabolic flux of glycolysis and had a positive effect on the synthesis of L-methionine.

## 5. Conclusions

In this study, the NADPH supply in *C. glutamicum* was increased by modifying the PPP pathway and heterologously expressing *gapC* and *pntAB*, thereby increasing the synthesis of L-methionine. In the constructed engineering strain YM6, the NADPH content reaches 2.51 nmol/10^6^ cell, which is 448.2% of the starting strain, and the L-methionine production reaches 0.64 g/L, which is 64.1% higher than that of the starting strain. Further ^13^C metabolic flux ratio analysis found that the flux ratio of G3P, DHAP, pyruvate, and other metabolites in glycolysis was significantly increased, which may provide a target for the efficient synthesis of L-methionine. However, the promotion of L-methionine production by simply increasing NADPH is limited, and systematic metabolic engineering is further work.

## Figures and Tables

**Table 1:** The Strains and plasmids used in this study.

Strains or Plasmids	Relevant Characteristics	Source
*E. coli* Top10	Wild type	Takara
*Clostrdium acetobutylicum* ATCC824	Wild type	ATCC
*C. glutamicun* ATCC13032	Wild type	ATCC
*C. glutamicun* YM1	*C. glutamicum* ATCC13032 chromosome with *zwf*^fbr^ gene	This study
*C. glutamicun* YM2	*C. glutamicum* ATCC13032 chromosome with *gnd*^fbr^ gene	This study
*C. glutamicun* YM3	*C. glutamicum* ATCC13032 chromosome with *zwf*^fbr^ and *gnd*^fbr^ gene	This study
*C. glutamicun* YM4	*C. glutamicum* ATCC13032 chromosome with *gapC* gene	This study
*C. glutamicun* YM5	*C. glutamicum* ATCC13032 chromosome with *pntAB* gene	This study
*C. glutamicun* YM6	*C. glutamicum* YM3 chromosome with *gapC* gene	This study
pK18*mobsacB*	pK18*mobsacB* non-replicating suicide plasmid	[9]
pLY-4	*E. coli* -*C. glutamicun* shuttle expression vector	[16]
pK18*mobsacB*-*zwf*^fbr^	pK18*mobsacB* carrying mutant *zwf* (G727A)	This study
pK18*mobsacB*-*gnd*^fbr^	pK18*mobsacB* carrying mutant *gnd*(T1083C)	This study
pLY-4-*gapC*	pLY-4 carrying *gapC* gene	This study
pLY-4-*pntAB*	pLY-4-pntAB carrying *pntAB* gene	This study

**Table 2:** Primers used in this study.

Name	Sequence (5′—3′)	Intention
zwf-F	CACAAGCTTATGGTGATCTTCGGTGTCACTGG	amplification of *zwf* gene
zwf-R	TATAAGCTTTTATGGCCTGCGCCAGGTGT	
gnd-F	CACGAATTCTTAAGCTTCAACCTCGGAGC′	amplification of *gnd* gene
gnd-R	GTGGAATTCATGCCGTCAAGTACGATCAA	
pntAB-F	CGAGGATCCATGCGAATTGGCATACCAAG	amplification of *pntAB* gene
pntAB-R	TCGGGATCCTTACAGAGCTTTCAGGATTG	
gapC-F	CGAGGATCCATGGCAAAGATAGCTATTAATG	amplification of *gapC* gene
gapC-R	TCGGGATCCCTATTTTGCTATTTTTGCA	
zwf-TBF	ACGTCCAGATCACCATGACTGAAGATATTGG	mutation of *zwf* gene
zwf-TBR	TCATGGTGATCTGGACGTGGTCAACGTA	
gnd-TBF	CGAGATCAAGGCTGGCCCCGACGAGAA	mutation of *gnd* gene
gnd-TBR	GGCCAGCCTTGATCTCGTCGAAGCCCT	
gndYZ-F	ATCGAAATCACCGCAGAGGTTC	validation of mutant *zwf* gene
gndYZ-R	GGAAGGAGCCATCCTTGTCGAT	
zwfYZ-F	ATGATGCAGCTTTCGACAACCT	validation of mutant *gnd* gene
zwfYZ-R	GATCTCTAAGGTACAAGCCGC	
zwfRT-F	TTGACCACGTCCAGATCACCATG	qRT-PCR of *zwf* gene
zwfRT-R	AGAGAGCACCTTGATCTTTTCTGC	
gndRT-F	GTTCTCTCCCAGGTGGATGCTG	qRT-PCR of *gnd* gene
gndRT-R	AAGTGCTTCCAGATCGGTGAGG	
pntABRT-F	CACCAACGCGATTTCAGGGAT	qRT-PCR of *pntAB* gene
pntABRT-R	ACCCCTTAATTTTTGCGGAAC	
gapCRT-F	GCATCATGCACAACTAACTGCTTAG	qRT-PCR of *gapC* gene
gapCRT-R	TGGCTTATAGCTTTAGCAGCAC	

**Table 3:** NADPH content and L-methionine production in different strains.

Strains	NADPH (nmol/10^6^ cell)	L-methionine (g/L)
*C. glutamicum* 13032	0.56 ± 0.01	0.39 ± 0.02
*C. glutamicum* YM1	0.94 ± 0.02	0.45 ± 0.03
*C. glutamicum* YM2	0.88 ± 0.02	0.46 ± 0.03
*C. glutamicum* YM3	1.41 ± 0.03	0.50 ± 0.04
*C. glutamicum* YM4	0.98 ± 0.02	0.58 ± 0.03
*C. glutamicum* YM5	1.06 ± 0.02	0.53 ± 0.01
*C. glutamicum* YM6	2.51 ± 0.02	0.64 ± 0.04

## Data Availability

The data presented in this study are available on request from the corresponding author. The data are not publicly available due to them containing information that could compromise researchers’ privacy/consent.
